# Neurobiology of human language and its evolution: primate and non-primate perspectives

**DOI:** 10.3389/fnevo.2013.00001

**Published:** 2013-01-28

**Authors:** Constance Scharff, Angela D. Friederici, Michael Petrides

**Affiliations:** ^1^Departments of Biology, Chemistry, and Pharmacy, Freie Universität BerlinBerlin, Germany; ^2^Department of Neuropsychology, Max Planck Institute for Human Cognitive and Brain SciencesLeipzig, Germany; ^3^Neurological Institute and HospitalMontreal, QC, Canada

The evolution of human language has been discussed for centuries from different perspectives. Linguistic theory has proposed grammar as a core part of human language that has to be considered in this context. Recent advances in neurosciences have allowed us to take a new neurobiological look on the similarities and dissimilarities of cognitive capacities and their neural basis across both closely and distantly related species. A couple of decades ago, the comparisons were mainly drawn between human and non-human primates, investigating the cytoarchitecture of particular brain areas and their structural connectivity. Moreover, comparative studies were conducted with respect to their ability to process grammars of different complexity. So far the available data suggest that non-human primates are able to learn simple probabilistic grammars, but not hierarchically structured complex grammars. The human brain, which easily learns both grammars, differs from the non-human brain (among others) in how two language-relevant brain regions (Broca's area in the inferior frontal cortex and the superior temporal cortex) are connected structurally by fiber tracts which run dorsally and ventrally in the primate brain. Whether the more dominant dorsal pathway in humans compared to non-human primates is causally related to this behavioral difference is an issue of current debate. Ontogenetic findings suggest at least a correlation between the maturation of the dorsal pathway and the behavior to process syntactically complex structures, although the ultimate causal prove is still not available. Thus, the neural basis of complex grammar processing in humans remains to be defined.

More recently it has been reported that songbirds are also able to distinguish between sound sequences reflecting complex grammar. Interestingly, songbirds learn to sing by imitating adult song in a process not unlike language development in children. Moreover, the neural circuits supporting this behavior in songbirds bear anatomical and functional similarities to those in humans. In adult humans the fiber tract connecting the auditory cortex and motor cortex dorsally is known to be involved in the repetition of spoken language. This pathway is present already at birth and is taken to play a major role during language acquisition. In songbirds, detailed information exist concerning the interaction of auditory, motor, and cortical-basal ganglia processing during song learning, and present a rich substrate for comparative studies.

The scope of the Research Topic was to bring together contributions of researchers from different fields, who investigate grammar processing in humans, non-human primates, and songbirds with the aim to find answers to the question of what constitutes the neurobiological basis of language and language learning.

A number of contributions discuss the ventral and dorsal pathways in human and non-human primates considering their functional roles in speech and language. Some of these take an evolutionary perspective comparing non-human and human primates (Rauschecker, 2012; Rilling et al., 2012), whereas other takes an ontogenetic perspective (Friederici, 2012). The functional roles of the ventral and dorsal pathways in language and other modalities in particular action including articulatory and hand gestures are discussed in further articles (Fitch, 2011; Aboitiz, 2012; Rijntjes et al., 2012). Two articles consider the language system at the interface of two other human specific abilities, namely number processing (Heim et al., 2012) and reading (Lachmann et al., 2012). A couple of contributions take the evolutionary perspective even further by including song birds into their comparative approach (Berwick et al., 2012; Kiggins et al., 2012; Petkov and Jarvis, 2012).

The selection of the articles provides a picture of the current views on the evolutionary and neurobiological basis of the language and language learning.
